# Eradication of *Enterococcus faecalis* Biofilms on Human Dentin

**DOI:** 10.3389/fmicb.2016.02055

**Published:** 2016-12-26

**Authors:** Eyal Rosen, Igor Tsesis, Shlomo Elbahary, Nimrod Storzi, Ilana Kolodkin-Gal

**Affiliations:** ^1^Department of Endodontology, Maurice and Gabriela Goldschleger School of Dental Medicine, Tel Aviv UniversityTel Aviv, Israel; ^2^Department of Molecular Genetics, Weizmann Institute of ScienceRehovot, Israel

**Keywords:** biofilms, dentin, viable but non-culturable (VBNC) state, *D*-amino acids, root canal therapy

## Abstract

**Objectives:** This work assesses different methods to interfere with *Enterococcus faecalis* biofilms formed on human dentin slabs.

**Methods:** First, methods are presented that select for small molecule inhibitors of biofilm targets using multi-well polystyrene biofilm plates. Next, we establish methodologies to study and interfere with biofilm formation on a medically relevant model, whereby biofilms are grown on human root dentin slabs.

**Results:** Non-conventional *D*-amino acid (*D*-Leucine) can efficiently disperse biofilms formed on dentin slabs without disturbing planktonic growth. Cation chelators interfere with biofilm formation on dentin slabs and polystyrene surfaces, and modestly impact planktonic growth. Strikingly, sodium hypochlorite, the treatment conventionally used to decontaminate infected root canal systems, was extremely toxic to planktonic bacteria, but did not eradicate biofilm cells. Instead, it induced a viable but non-culturable state in biofilm cells when grown on dentin slabs.

**Conclusion:** Sodium hypochlorite may contribute to bacterial persistence. A combination of the methods described here can greatly contribute to the development of biofilm inhibitors and therapies to treat *Enterococcus faecalis* infections formed in the root canal system.

## Introduction

Bacterial biofilms are multicellular microbial communities that adhere to surfaces and interfaces (Kolter and Greenberg, 2006). The formation and maintenance of biofilms is dependent on the production of extracellular substances including proteins and exopolysaccharides that constitute the extracellular matrix. These extracellular matrices secure the bacteria together in a multicellular community (Parsek and Singh, 2003; Branda et al., 2005; Oppenheimer-Shaanan et al., 2013; Vlamakis et al., 2013). Biofilms offer the microbial communities shelter from environmental insults and assaults, attachment to a host and access to oxygen and nutrients (Costerton et al., 1987; Chen et al., 2012).

Microbial biofilms account for over 80% of microbial infections in the body (Costerton et al., 1999; Stewart, 2002; Mah et al., 2003; Fux et al., 2005; Oppenheimer-Shaanan et al., 2013), and are considered as a primary cause of apical periodontitis in teeth with infected root canal spaces (Haapasalo and Shen, 2012). Apical periodontitis is a relatively common dental pathology that involves an inflammatory reaction and destruction of tissues around the apex of a tooth-root. This is caused by microbial invasion and infection of the dental pulp, and biofilm colonization within the root canal system (Ricucci and Siqueira, 2010).

Gram-positive and facultative anaerobes are the most frequently isolated species within treated canals in teeth with persistent intra-radicular infections, with *Enterococcus faecalis (E. faecalis)* being the most prevalent (Zhang et al., 2015). In the root canal environment, E. *faecalis* bacterium plays an important role in bacterial biofilm formation, and therefore *E. faecalis* biofilms are considered to be an appropriate model for testing novel antimicrobial treatments (Meire et al., 2012; Du et al., 2013; Tay et al., 2015; Shlezinger et al., 2016).

One of the primary goals of root canal treatment is to eliminate bacteria from the root canal system in order to treat or prevent apical periodontitis (Kishen, 2012). However, bacteria within biofilm communities are 10-fold to 1000-fold more resistant to antimicrobial agents and antibiotics than planktonic (free-living) bacteria, and are also able to effectively evade the immune system (Costerton et al., 1999; Stewart, 2002; Mah et al., 2003; Fux et al., 2005; Oppenheimer-Shaanan et al., 2013; Tay et al., 2015). For these reasons bacterial biofilms pose a major obstacle to endodontic disinfection in root canal systems, and therefore methods to promote biofilm dispersal may ultimately improve the treatment outcome (Kishen, 2012; Meire et al., 2012; Du et al., 2013).

The key element in the elimination of intra-canal biofilms is the use of anti-microbial irrigating solutions during the root canal treatment. However, currently the most commonly used anti-microbial irrigation solution, sodium hypochlorite, has a limited ability to completely eliminate the biofilm from the root canal, sometimes leading to persistent infection. Thus, stressing the need to develop novel anti-microbial biofilm agents in order to achieve predictable, effective disinfection of the root canal system (Ricucci and Siqueira, 2010).

Small molecules that target the cell envelope were found to be efficient inhibitors for biofilms formed by Gram-positive bacteria, and to effectively disperse the biofilms. Recently, flavomycin, an antibiotic that inhibits transglycosylation directly by binding the transglycosylation domain of PBP enzymes (Dengler et al., 2011) was found to antagonize biofilm formation but not planktonic growth in the soil bacterium, *Bacillus subtilis* (Bucher et al., 2015). An additional group of cell-wall interfering agents that promote dispersal are non-canonical *D*-amino acids (Bucher et al., 2015, 2016). *D*-amino acids compete with *D*-Alanine for the fifth position in the *B. subtilis* pentapeptide, and interfere with transpeptidation (Lam et al., 2009; Cava et al., 2011; Lupoli et al., 2011) and transglycosylation (Lam et al., 2009). *D*-amino acids were found to inhibit and disperse biofilms without affecting planktonic growth in various model organisms (Kolodkin-Gal et al., 2010; Hochbaum et al., 2011; Yu et al., 2012; Sanchez et al., 2013; Li and Wang, 2014; Bucher et al., 2015, 2016; She et al., 2015), but to the best of our knowledge their efficacy on endodontic biofilms was never evaluated.

An additional method to induce dispersal of biofilms in various model organisms is the use of cation chelators. Iron is an essential component of many metabolically relevant proteins in living cells, and the maintenance of biofilms requires higher concentrations of iron than planktonic growth (Banin et al., 2005; Ramos et al., 2010; Kolodkin-Gal et al., 2013). The functional siderophore pyoverdin is required for biofilm maturation of *P. aeruginosa*, and its absence promotes disassembly (Banin et al., 2005). Moreover, lactoferrin, an innate immunity protein, was shown to disrupt *P. aeruginosa* biofilm formation by sequestering Fe(III) from siderophores (Singh et al., 2002), and chelation of cations by Ethylenediaminetetraacetic acid (EDTA) was demonstrated to disperse *staphylococcal* biofilms (Raad et al., 2003). Overall, chelation of cations in biofilm deformation could result in effective therapeutic strategies for eradication of medical biofilms. In root-canal treatment, EDTA is traditionally used as a chelating agent to remove calcium, demineralize and soften dentin, and to remove the “smear layer,” a surface film of debris containing dentin particles, remnants of vital or necrotic pulp tissue, and bacterial components, retained on the dentin and other surfaces after the root canal procedure (de Almeida et al., 2016).

The aim of this study was to evaluate the use of small molecules that were previously shown to inhibit and eradicate biofilms, for the elimination of *E. faecalis* biofilms grown on human dentin slabs, and compare their efficiency with sodium hypochlorite, a commonly used antimicrobial agent in root canal treatment.

## Materials and Methods

### Samples Preparation

Twenty freshly extracted single rooted fully developed intact human teeth were stored in 0.05% sodium hypochlorite solution. Informed consent was obtained from the extracted teeth donors.

This study was approved by the Tel Aviv university ethics committee.

The crowns of the selected teeth were removed in order to obtain multiple root specimens of 13 mm length, and the apical 3 mm of the root end was resected without a bevel using Zakaria high speed bur (Maillefer, Ballaigues, Switzerland). The root canal lumen was then enlarged to a minimum of 0.5 mm using low speed burs (Gates Glidden Drills, Dentsply Maillefer, Tulsa, OK, USA). The roots were embedded in self-cure acrylic repair material (UNIFAST Trad, GC America). To prepare the dentin slabs, the roots were cut perpendicular to the long axis of the root under water cooling with a diamond saw rotating at 500 rpm (Isomet, Buehler Ltd., Lake Bluff, IL, USA). Two dentin slabs of 1 mm thickness each were obtained from each root (Kuci et al., 2014), see Supplementary Figure S1. The specimens were then placed in small dishes and sterilized overnight using ethylene oxide gas (Brosco et al., 2010). For each of the indicated treatment at least nine independent dentin slabs were evaluated under the same conditions.

### Strains and Media

All of the experiments were performed in a clinical isolate of *Enterococcus faecalis* 29212 (Minogue et al., 2014). To confirm reproducible results we evaluated biofilm formation of a single virulent strain on-top of artificial and biological surfaces.

The strains were routinely manipulated in LB broth (Difco), or in TSB broth (Difco), enriched with 0.5% glucose (Sigma) (Lopez and Kolter, 2010).

### Biofilm Formation Assay

Cells were grown in LB from a single colony isolated over LB plates to a mid-logarithmic phase of growth (6 h at 37°C with shaking). To grow biofilms, 1.5 μl of starter culture was inoculated into the TSB glucose media in 96-well polystyrene plates and further incubated for 24 h. The growth media were either applied or not with one of the following substances: (i) A Sodium hypochlorite was added to the final concentration of 0.6% from a stock solution of sodium hypochlorite solution, reagent grade, 10–15% (sigma), (ii) *D*-Leucine (Sigma–Aldrich) was added to the final concentration of 2 mM from a stock solution of 76 mM *D*-Leucine in DDW, (iii) flavomycin (AK Scientific) was added to the final concentration of 2 μg/ml from a stock solution of 2 mg/ml (iv) EDTA (Sigma–Aldrich) was added to the final concentration of 0.5 mM from a stock solution of 50 mM (0.5 mM), and (v) 2-2′ bipyridyl (Sigma–Aldrich) was added to a final concentration of 10 μg/ml from a stock solution of 10 mg/ml in ethanol. In the case of the 96-well polystyrene plate, the crystal violet (Sigma–Aldrich) assay was performed as described by Friedman and Kolter (2004).

### Growth Measurements

Cells were grown from a single colony isolated from LB plates to a stationary phase of growth (12 h at 37°C with shaking). The culture was then diluted 1:25 in 5 ml liquid TSB glucose medium (Thermo Scientific). Cells were grown with agitation at 37°C for 10 h in a growth chamber, and the optical density at 600 nm (OD_600_) was measured every 2 h. Cells were either grown in presence or absence of cell-wall inhibiting molecules, as indicated in the corresponding figure legend.

### Determination of Cell Density and Live Cell Counts during Dentin Disk Colonization

Cells were grown in LB from a single colony isolated over LB plates to a mid-logarithmic phase of growth (6 h at 37°C with shaking). To grow biofilms, 1.5 μl of starter culture was inoculated into 6 ml TSB glucose media dispensed into a Petri dish containing the dentin disks. Cells were grown on-top of dentin disks as described above. Following 24 h of growth, the media was removed from the dentin disks, and the associated bacteria were incubated in different substances and treated further, as specified in the legends for each figure. Following incubation, the suspension solution was collected and further evaluated for the live cell counts as well as the biofilm fraction. The biofilm fraction was obtained by three washes of the dentin disks with phosphate buffer to the final volume of the suspension solution. To determine the number of live cells, cells were serially diluted in phosphate-buffered saline (PBS; Biological Industries, Israel), plated on LB plates, and colony forming units (CFU) were counted after incubation at 37°C overnight as done by us previously (Bucher et al., 2015).

### Confocal Scanning Laser Microscopy and Live∖Dead Evaluation

To determine culture density and live cell counts of cells grown on the disks, cells were harvested from a dentin biofilm dispersal assay (described above): Cells were grown on-top of dentin disks as described above. Following 24 h of growth, the media was removed from the dentin disks, and the associated bacteria were incubated in different substances and treated further, as specified in the legends for each figure. Samples of biofilms grown for 24 h and treated as indicated were stained using LIVE/DEAD BacLight Bacterial Viability kit L-7012 for microscopy and quantitative assays (Molecular Probes, Eugene, OR, USA) containing separate vials of the two component dyes (SYTO 9 and propidium iodide in 1:1 mixture) in solution was used for staining of the biofilm following the manufacturer’s instructions. The excitation/emission maxima for these dyes is approximately 480–500 nm for the SYTO 9 stain and 490–635 nm for propidium iodide (Aziz et al., 2010). Fluorescence from the stained cell was viewed under a confocal laser scanning microscope (Leica TCS SP5, Leica Microsystems CMS GmbH Germany). Single channel and simultaneous dual-channel imaging was used to display green and red fluorescence (Zapata et al., 2008). Confocal laser scanning microscope images of the biofilms were acquired by the LAS AF software (version 2.6.0.7266; Leica Microsystems CMS GmbH) at a resolution of 512 × 512 pixels. The mounted specimens were observed using a X4 lens. Confocal LIVE/DEAD images were analyzed and quantitated using the above mentioned software (LAS AF; Zapata et al., 2008; Shen et al., 2010; Kuci et al., 2014). The specimens were coded for blind evaluation.

### Statistical Methods

All studies were performed in duplicates or triplicates at least three separate and independent times. Data are expressed as average values ± standard deviations of the means. Parametric testing was performed after confirming that raw data were normally distributed. Data were analyzed by student’s *t*-test, used to determine if the set of treated versus the untreated control are different from each other (A paired *t*-test comparing two sets of measurements) differ and *P* values of less than 0.1 were considered significant.

## Results

### Evaluation of Biofilm Inhibitors on Planktonic Growth and Biofilm Formation on Polystyrene Surfaces

**FIGURE 1:**
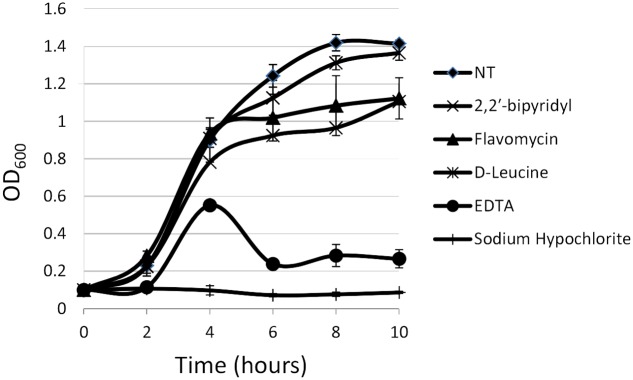
**Analyzing the effects of small molecules on planktonic growth of *Enterococcus faecalis***. Growth of strain 29212 was assessed at 37°C with shaking in liquid TSB-glucose medium (untreated) or applied with the following: Sodium hypochlorite (0.6%), *D*-Leucine (2 mM), flavomycin (2 μg/ml), EDTA (0.5 mM), and 2-2′ bipyridyl (10 μg/ml). Growth was monitored by measuring OD_600_. Results are averages of three independent experiment performed in duplicates and their standard deviations.

Systematic evaluation of small molecule biofilm inhibitors was performed on planktonic growth (**Figure 1**) and biofilm formation, according to the Microtiter Dish Biofilm Formation Assay (Friedman and Kolter, 2004). Three categories of biofilm inhibitors were tested: (i) Small molecules that target the cell envelope: Flavomycin, an antibiotic that inhibits transglycosylation directly by binding the transglycosylation domain of PBP enzymes (Dengler et al., 2011); and *D*-Leucine, a non-canonical *D*-amino acid, which competes with *D*-Alanine for the fifth position in the *B. subtilis* pentapeptide (Bucher et al., 2015), and interferes with transpeptidation (Lam et al., 2009; Cava et al., 2011; Lupoli et al., 2011) and transglycosylation (Lam et al., 2009). (ii) Cation chelators: EDTA, a chelating agent that sequesters a variety of polyvalent cations such as calcium; and 2,2′-bipyridyl, an organic bidentate chelating ligand, forming complexes with many transition metals, with a strong affinity to iron. (iii) Sodium hypochlorite (bleach) – commonly used concentrations between 0.5 and 6% for irrigation in root canal treatments (Bystrom and Sundqvist, 1985; Gomes et al., 2001; Haapasalo et al., 2014).

For planktonic growth, sodium hypochlorite turned out to be most toxic, eliminating *E. faecalis* growth altogether (**Figure 1**), EDTA, flavomycin and 2-2′ bipyridyl inhibited (reversibly) bacterial growth at indicated concentrations, and *D*-Leucine at concentrations of up to 2 mg/ml had little or no effect on planktonic growth.

In contrast, the results from the assay on biofilm formation in polystyrene wells were as following: the mildly toxic 2-2′ bipyridyl and flavomycin had a modest inhibitory effect on biofilm formation, while EDTA (mildly toxic), *D*-Leucine (non-toxic), and sodium hypochlorite (highly toxic) inhibited biofilm formation comparably and significantly. These results indicate that inhibition of biofilm formation may not be directly correlated to inhibition of planktonic growth.

### Evaluation of Biofilm Inhibitors *Enterococcus faecalis* Biofilms Formed on a Root-Dentin Model

**FIGURE 2:**
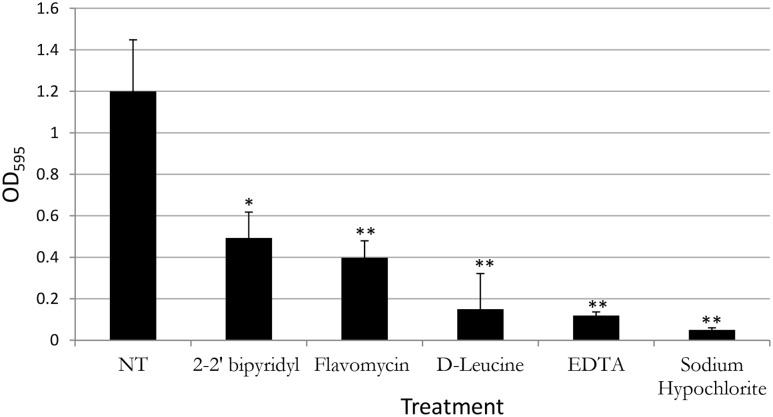
**Analyzing the effects of small molecules on biofilm formation of *E. faecalis* in a microplate model**. Single colony of Strain 29212 was grown at 37°C with shaking in liquid TSB-glucose medium to a mid-logarithmic stage. Cells were diluted 1:100 into a fresh medium (untreated) or applied with the following small molecules: Sodium hypochlorite (0.6%), *D*-Leucine (2 mM), flavomycin (2 μg/ml), EDTA (0.5 mM), and 2-2′ bipyridyl (10 μg/ml). Cultures were split into a 96-well polystyrene plate, 100 μL in each well, and further incubated at 37°C for 24 h. Biofilm formation was assessed by crystal violet staining as described in “Material and Methods.” Results are averages of three independent experiments performed with five repeats. *P* value was calculated using a student’s *t*-test. ^∗^*P* < 0.1, ^∗∗^*P* < 0.05, compared with the untreated control.

**FIGURE 3:**
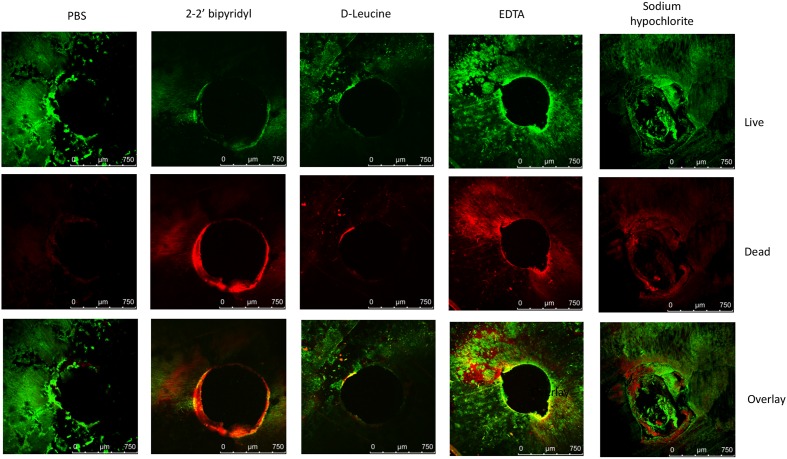
**Analyzing the effects of small molecules on pre-established biofilms of *E. faecalis* on human dentin disks**. Single colony of Strain 29212 was grown at 37°C with shaking in liquid TSB-glucose medium to a mid-logarithmic stage. Cells were diluted 1:100 into fresh medium in polystyrene plates, containing a fixed dentin disk. Following 24 h of incubation cells were applied with the following solutions, phosphate-buffered saline (PBS), or a PBS solution applied with the following substances: Sodium hypochlorite (0.6%), *D*-Leucine (2 mM), flavomycin (2 μg/ml), EDTA (0.5 mM), and 2-2′ bipyridyl (10 μg/ml) for 2 h. Cells were washed, stained with BacLight Bacterial Viability kit and imaged as described in the section “Materials and Methods.” ^∗^*P* < 0.1, ^∗∗^*P* < 0.05, compared with the PBS treatment.

**FIGURE 4:**
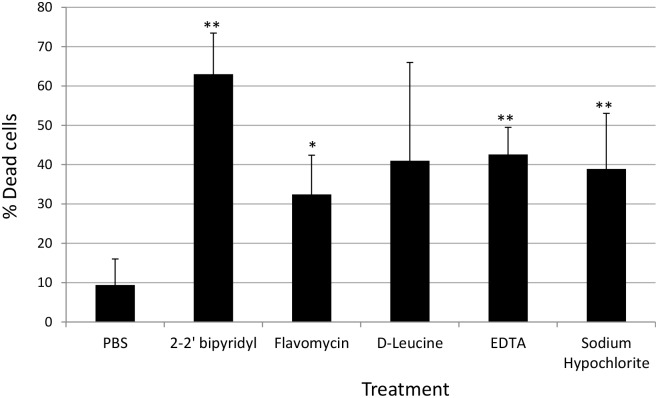
**Analyzing the effects of small molecules on the viability of *E. faecalis* biofilm cells grown on human dentin disks**. Single colony of Strain 29212 was grown at 37°C with shaking in liquid TSB-glucose medium to a mid-logarithmic stage. Cells were diluted 1:100 into a fresh medium in polystyrene plates, containing a fixed dentin disk. Following 24 h of incubation cells were applied with the following solutions, PBS, or a PBS solution applied with the following substances: Sodium hypochlorite (0.6%), *D*-Leucine (2 mM), flavomycin (2 μg/ml), EDTA (0.5 mM), and 2-2′ bipyridyl (10 μg/ml) for 2 h. Cells were washed and imaged as described in the section “Materials and Methods.” The number of cells stained in PI (Dead), and the number of cells stained with fluorescein was calculated as described in the section “Materials and Methods.” Results are averages of two independent experiments performed with at least three repeats. *P* value was calculated using a student’s *t*-test. ^∗^*P* < 0.1, ^∗∗^*P* < 0.05, compared with the PBS treatment.

In order to establish a more ecological dentin model, root dentin slabs of 1 mm thickness each were cut as described in the section “Materials and Methods” (Supplementary Figure S1). The sterile dentin disk was then inoculated with *E. faecalis* and further incubated in biofilm media. As shown, *E. faecalis* cells formed a thick biofilm on the dentin slab within 24 h. Once a biofilm was established, we used the several biofilm inhibitors that proved to effectively inhibit biofilm formation on-top of polystyrene plates (**Figure 2**) and evaluated their effect on dispersing root-dentin associated biofilms. For this purpose, we first scored the remaining biofilm using the Live/Dead BacLight Viability Kit (**Figure 3**). The outcome of the application of biofilm inhibitors to an established dentin-associated biofilm differed dramatically between treatments. Surprisingly, the sodium hypochlorite treatment, found to be most toxic to planktonic growth, had little effect on removal of the biofilm biomass, and only modestly impacted the overall viability of the biofilm cells. The concentration of flavomycin which halted planktonic growth and biofilm formation (**Figures 1** and **2**) had no impact on established biofilms (Supplementary Figure S2), and chelation of cations by EDTA had little or no effect on the overall biofilm’s biomass. However, EDTA treatment significantly increased the proportion of dead cells (**Figures 3** and **4**). Specific chelation of iron by 2-2′ bipyridyl greatly increased cell death within dentin-associated biofilms (**Figures 3** and **4**). The most efficient treatment was *D*-Leucine, as it significantly dispersed the biofilm’s biomass (**Figure 3**) and modestly increased the fraction of dead cells compared with the control. Importantly, the *D*-Leucine treatment was found to be effective in sub-toxic concentration compatible with endodontic therapy.

**FIGURE 5:**
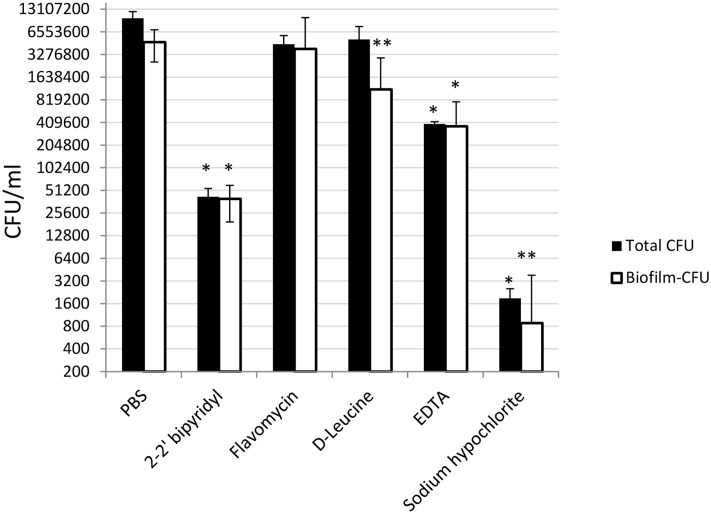
**Analyzing the effects of small molecules on the cultivability of *E. faecalis* biofilm cells grown on human dentin disks**. Single colony of Strain 29212 was grown at 37°C with shaking in liquid TSB-glucose medium to a mid-logarithmic stage. Cells were diluted 1:100 into a fresh medium in polystyrene plates, containing a fixed dentin disk. Following 24 h of incubation, cells were applied with the following solutions, PBS, or a PBS solution applied with the following substances: Sodium hypochlorite (0.6%), *D*-Leucine (2 mM), flavomycin (2 μg/ml), EDTA (0.5 mM), and 2-2′ bipyridyl (10 μg/ml) for 2 h. Cells were then obtained by rigorous pipetting and cultures as described in the section “Materials and Methods.” Results are averages of two independent experiments performed with at-least four repeats. ^∗^*P* < 0.1, ^∗∗^*P* < 0.05, compared with the PBS treatment.

To further evaluate the viability of the *E. faecalis* following different treatments, we assessed the replicative cell counts from the treated biofilms and the growth media. Strikingly, though the conservation of the biofilm biomass following sodium hypochlorite treatment was evident between different experiments (**Figure 3**), very few culturable cells could be eluted from the dentin disks and the inoculation media (**Figure 5**). In contrast, replicative cell counts from other treatments correlated better with the confocal examination. This result could be an indication that the sodium hypochlorite treatment is promoting a viable but not culturable state (VBNC) in root-associated biofilms.

## Discussion

*Enterococcus faecalis* is a commensal Gram-positive microorganism residing within the gastrointestinal tract. Nonetheless, it can cause life-threatening infections such as endocarditis, bacteremia, urinary tract infection, and meningitis (Khalifa et al., 2015), and is especially problematic in hospitals where antibiotic resistance is developed (Deshpande et al., 2007). In addition, *E. faecalis* is frequently recovered from persistent infections associated with root canal treatment failures (Zapata et al., 2008), and can result in chronic or acute inflammation and destruction of the tissues surrounding the tip of the tooth-root with subsequent development of abscesses. Despite meticulous mechanical and chemical preparation during root canal treatment, infection may persist (Zhang et al., 2015), in most of the treated and filled root canals, and in some cases may lead to treatment failure and further complications (Molander et al., 1998). To date, the available therapeutic tools to efficiently and predictably eradicate intra-canal *E. faecalis* biofilm infection are limited (Paganelli et al., 2012). Biofilms may pose a severe health threat, since at this phase bacteria become inaccessible to antibacterial agents and the body’s immune system (Bryers, 2008; Wang et al., 2012). The penetration failure may be associated with various factors, including the extracellular matrix encapsulating the biofilm cells, and multidrug resistance development of bacteria within the biofilm (Bryers, 2008).

In this study several biofilm inhibitors and dispersing agents were evaluated for their ability to combat *E. faecalis* infection on dentin slabs mimicking *E. faecalis* root canal infections. Surprisingly, sodium hypochlorite, the commonly used anti-bacterial irrigation solution for treatment of root canal infections failed to reduce the biofilm biomass on dentin disks (**Figure 3**), though it was most efficient in reducing the replicative properties of the biofilm’s cells (**Figure 5**).

In addition, our results may imply the induction of a VBNC state in *enterococcus* biofilms treated with sodium hypochlorite. The entry of bacteria into a state described as VBNC has been reported repeatedly for a large number of bacterial species, and among them several Gram-positive bacteria, including bacteria that reside in the oral cavity (Hiyari and Bennett, 2011; E et al., 2015). A bacterium in the VBNC state has been defined as a cell which can be demonstrated to be metabolically active, while being incapable of undergoing the sustained cellular division required for growth in or on a medium normally supporting growth of that cell (Koch, 1997). Importantly, the presence of *E. faecalis* on dentin slabs following a treatment with sodium hypochlorite may explain the resistance of *E. faecalis* biofilms to the currently used treatment protocols, and involvement in treatment failure with persistent infections following root canal treatments (Zhang et al., 2015).

In contrast, the anti-biofilm treatment *D*-Leucine efficiently dispersed dentin-associated biofilms with little effect on the viability of the biofilm cells. The biocompatibility of *D*-amino acids is especially promising as they were non-cytotoxic to human osteoblasts at concentrations less than 50 mmol/L, 25 times more than the required concentration for biofilm inhibition (Harmata et al., 2015) and were non-toxic when orally administrated (Tsume et al., 2014). Inducing dispersal by a sub-toxic concentrations of an anti-biofilm agent is of high interest (Kolodkin-Gal et al., 2010; Romero and Kolter, 2011; Bucher et al., 2015, 2016), as it is expected to reduce the selective pressure for the success of resistant mutants. Indeed toxic concentrations of *D*-amino acids were shown to select for mutants that carry various alleles of resistance (Leiman et al., 2015).

The iron chelator 2-2′ bipyridyl efficiently induced cell death within dentin-associated biofilms, but did not change the overall dentin-associated biomass. 2-2′ bipyridyl can inhibit Fe^2+^ containing enzymes at 10^-8^ M. However, in the concentrations used in our study it is a widely used ligand (Kaes et al., 2000), and may be appropriate for endocarditis treatment. Thus, our overall result highlights the need and the potential for combination therapies in root canal biofilm infections.

## Author Contributions

IK-G, ER, and IT designed experiments. NS, SE, and IK-G performed the experiments. NS, SE, and IK-G contributed materials and reagents. IK-G, SE, ER, and IT wrote the paper.

## Conflict of Interest Statement

The authors declare that the research was conducted in the absence of any commercial or financial relationships that could be construed as a potential conflict of interest. The reviewer TMBR and handling Editor declared their shared affiliation and the handling Editor states that the process nevertheless met the standards of a fair and objective review.

## References

[B1] Aziz A. Parmar D. Mcnaughton A. Tompkins G. R. (2010). Bacterial viability determination in a dentinal tubule infection model by confocal laser scanning microscopy. *Methods Mol. Biol.* 666 141–150. 10.1007/978-1-60761-820-1_10 20717783

[B2] Banin E. Vasil M. L. Greenberg E. P. (2005). Iron and *Pseudomonas aeruginosa* biofilm formation. *Proc. Natl. Acad. Sci. U.S.A.* 102 11076–11081. 10.1073/pnas.0504266102 16043697 PMC1182440

[B3] Branda S. S. Vik S. Friedman L. Kolter R. (2005). Biofilms: the matrix revisited. *Trends Microbiol.* 13 20–26. 10.1016/j.tim.2004.11.006 15639628

[B4] Brosco V. H. Bernardineli N. Torres S. A. Consolaro A. Bramante C. M. De Moraes I. G. (2010). Bacterial leakage in obturated root canals-part 2: a comparative histologic and microbiologic analyses. *Oral Surg. Oral Med. Oral Pathol. Oral Radiol. Endod.* 109 788–794. 10.1016/j.tripleo.2009.11.036 20416539

[B5] Bryers J. D. (2008). Medical biofilms. *Biotechnol. Bioeng.* 100 1–18. 10.1002/bit.21838 18366134 PMC2706312

[B6] Bucher T. Kartvelishvily E. Kolodkin-Gal I. (2016). Methodologies for studying *B. subtilis* biofilms as a model for characterizing small molecule biofilm inhibitors. *J. Vis. Exp.* 10.3791/54612 PMC5092178 27768058

[B7] Bucher T. Oppenheimer-Shaanan Y. Savidor A. Bloom-Ackermann Z. Kolodkin-Gal I. (2015). Disturbance of the bacterial cell wall specifically interferes with biofilm formation. *Environ. Microbiol. Rep.* 7 990–1004. 10.1111/1758-2229.12346 26472159

[B8] Bystrom A. Sundqvist G. (1985). The antibacterial action of sodium hypochlorite and EDTA in 60 cases of endodontic therapy. *Int. Endod. J.* 18 35–40. 10.1111/j.1365-2591.1985.tb00416.x 3922900

[B9] Cava F. De Pedro M. A. Lam H. Davis B. M. Waldor M. K. (2011). Distinct pathways for modification of the bacterial cell wall by non-canonical D-amino acids. *EMBO J.* 30 3442–3453. 10.1038/emboj.2011.246 21792174 PMC3160665

[B10] Chen Y. Cao S. Chai Y. Clardy J. Kolter R. Guo J. H. (2012). A *Bacillus subtilis* sensor kinase involved in triggering biofilm formation on the roots of tomato plants. *Mol. Microbiol.* 85 418–430. 10.1111/j.1365-2958.2012.08109.x 22716461 PMC3518419

[B11] Costerton J. W. Cheng K. J. Geesey G. G. Ladd T. I. Nickel J. C. Dasgupta M. (1987). Bacterial biofilms in nature and disease. *Annu. Rev. Microbiol.* 41 435–464. 10.1146/annurev.mi.41.100187.002251 3318676

[B12] Costerton J. W. Stewart P. S. Greenberg E. P. (1999). Bacterial biofilms: a common cause of persistent infections. *Science* 284 1318–1322. 10.1126/science.284.5418.1318 10334980

[B13] de Almeida J. Hoogenkamp M. Felippe W. T. Crielaard W. Van Der Waal S. V. (2016). Effectiveness of EDTA and modified salt solution to detach and kill cells from *Enterococcus faecalis* biofilm. *J. Endod.* 42 320–323. 10.1016/j.joen.2015.11.017 26723483

[B14] Dengler V. Meier P. S. Heusser R. Berger-Bachi B. Mccallum N. (2011). Induction kinetics of the *Staphylococcus aureus* cell wall stress stimulon in response to different cell wall active antibiotics. *BMC Microbiol.* 11:16 10.1186/1471-2180-11-16 PMC3032642 21251258

[B15] Deshpande L. M. Fritsche T. R. Moet G. J. Biedenbach D. J. Jones R. N. (2007). Antimicrobial resistance and molecular epidemiology of vancomycin-resistant enterococci from North America and Europe: a report from the SENTRY antimicrobial surveillance program. *Diagn. Microbiol. Infect. Dis.* 58 163–170. 10.1016/j.diagmicrobio.2006.12.022 17368801

[B16] Du T. Shi Q. Shen Y. Cao Y. Ma J. Lu X. (2013). Effect of modified nonequilibrium plasma with chlorhexidine digluconate against endodontic biofilms in vitro. *J. Endod.* 39 1438–1443. 10.1016/j.joen.2013.06.027 24139270

[B17] E J. Jiang Y. T. Yan P. F. Liang J. P. (2015). Biological changes of *Enterococcus faecalis* in the viable but nonculturable state. *Genet. Mol. Res.* 14 14790–14801. 10.4238/2015.November.18.44 26600540

[B18] Friedman L. Kolter R. (2004). Genes involved in matrix formation in *Pseudomonas aeruginosa* PA14 biofilms. *Mol. Microbiol.* 51 675–690. 10.1046/j.1365-2958.2003.03877.x 14731271

[B19] Fux C. A. Costerton J. W. Stewart P. S. Stoodley P. (2005). Survival strategies of infectious biofilms. *Trends Microbiol.* 13 34–40. 10.1016/j.tim.2004.11.010 15639630

[B20] Gomes B. P. F. A. Ferraz C. C. R. Vianna M. E. Berber V. B. Teixeira F. B. Souza F. J. (2001). In vitro antimicrobial activity of several concentrations of sodium hypochlorite and chlorhexidine gluconate in the elimination of *Enterococcus faecalis*. *Int. Endod. J.* 34 424–428. 10.1046/j.1365-2591.2001.00410.x 11556507

[B21] Haapasalo M. Shen Y. Wang Z. Gao Y. (2014). Irrigation in endodontics. *Br. Dent. J.* 216 299–303. 10.1038/sj.bdj.2014.204 24651335

[B22] Haapasalo M. Shen Y. A. (2012). Current therapeutic options for endodontic biofilms. *Endod. Topics* 22 79–98. 10.1111/j.1601-1546.2012.00281.x

[B23] Harmata A. J. Ma Y. Sanchez C. J. Zienkiewicz K. J. Elefteriou F. Wenke J. C. (2015). D-amino acid inhibits biofilm but not new bone formation in an ovine model. *Clin. Orthop. Relat. Res.* 473 3951–3961. 10.1007/s11999-015-4465-9 26201421 PMC4626484

[B24] Hiyari S. Bennett K. M. (2011). Dental diagnostics: molecular analysis of oral biofilms. *J. Dent. Hyg.* 85 256–263. 22309866

[B25] Hochbaum A. I. Kolodkin-Gal I. Foulston L. Kolter R. Aizenberg J. Losick R. (2011). Inhibitory effects of D-amino acids on *Staphylococcus aureus* biofilm development. *J. Bacteriol.* 193 5616–5622. 10.1128/JB.05534-11 21856845 PMC3187230

[B26] Kaes C. Katz A. Hosseini M. W. (2000). Bipyridine: the most widely used ligand. A review of molecules comprising at least two 2,2′-bipyridine units. *Chem. Rev.* 100 3553–3590. 11749322 10.1021/cr990376z

[B27] Khalifa L. Brosh Y. Gelman D. Coppenhagen-Glazer S. Beyth S. Poradosu-Cohen R. (2015). Targeting *Enterococcus faecalis* biofilms with phage therapy. *Appl. Environ. Microbiol.* 81 2696–2705. 10.1128/AEM.00096-15 25662974 PMC4375334

[B28] Kishen A. (2012). Advanced therapeutic options for endodontic biofilms. *Endod. Topics* 22 99–123. 10.1111/j.1601-1546.2012.00284.x

[B29] Koch A. L. (1997). Microbial physiology and ecology of slow growth. *Microbiol. Mol. Biol. Rev.* 61 305–318. 9293184 10.1128/mmbr.61.3.305-318.1997 PMC232613

[B30] Kolodkin-Gal I. Elsholz A. K. Muth C. Girguis P. R. Kolter R. Losick R. (2013). Respiration control of multicellularity in *Bacillus subtilis* by a complex of the cytochrome chain with a membrane-embedded histidine kinase. *Genes Dev.* 27 887–899. 10.1101/gad.215244.113 23599347 PMC3650226

[B31] Kolodkin-Gal I. Romero D. Cao S. Clardy J. Kolter R. Losick R. (2010). D-amino acids trigger biofilm disassembly. *Science* 328 627–629. 10.1126/science.1188628 20431016 PMC2921573

[B32] Kolter R. Greenberg E. P. (2006). Microbial sciences: the superficial life of microbes. *Nature* 441 300–302. 10.1038/441300a 16710410

[B33] Kuci A. Alacam T. Yavas O. Ergul-Ulger Z. Kayaoglu G. (2014). Sealer penetration into dentinal tubules in the presence or absence of smear layer: a confocal laser scanning microscopic study. *J. Endod.* 40 1627–1631. 10.1016/j.joen.2014.03.019 25260735

[B34] Lam H. Oh D. C. Cava F. Takacs C. N. Clardy J. De Pedro M. A. (2009). D-amino acids govern stationary phase cell wall remodeling in bacteria. *Science* 325 1552–1555. 10.1126/science.1178123 19762646 PMC2759711

[B35] Leiman S. A. Richardson C. Foulston L. Elsholz A. K. First E. A. Losick R. (2015). Identification and characterization of mutations conferring resistance to D-amino acids in *Bacillus subtilis*. *J. Bacteriol.* 197 1632–1639. 10.1128/JB.00009-15 25733611 PMC4403649

[B36] Li J. Wang N. (2014). Foliar application of biofilm formation-inhibiting compounds enhances control of citrus canker caused by *Xanthomonas citri* subsp. citri. *Phytopathology* 104 134–142. 10.1094/PHYTO-04-13-0100-R 23901828

[B37] Lopez D. Kolter R. (2010). Functional microdomains in bacterial membranes. *Genes Dev.* 24 1893–1902. 10.1101/gad.1945010 20713508 PMC2932971

[B38] Lupoli T. J. Tsukamoto H. Doud E. H. Wang T. S. Walker S. Kahne D. (2011). Transpeptidase-mediated incorporation of D-amino acids into bacterial peptidoglycan. *J. Am. Chem. Soc.* 133 10748–10751. 10.1021/ja2040656 21682301 PMC3172152

[B39] Mah T. F. Pitts B. Pellock B. Walker G. C. Stewart P. S. O’toole G. A. (2003). A genetic basis for *Pseudomonas aeruginosa* biofilm antibiotic resistance. *Nature* 426 306–310. 10.1038/nature02122 14628055

[B40] Meire M. A. Coenye T. Nelis H. J. De Moor R. J. (2012). Evaluation of Nd:YAG and Er:YAG irradiation, antibacterial photodynamic therapy and sodium hypochlorite treatment on *Enterococcus faecalis* biofilms. *Int. Endod. J.* 45 482–491. 10.1111/j.1365-2591.2011.02000.x 22243483

[B41] Minogue T. D. Daligault H. E. Davenport K. W. Broomall S. M. Bruce D. C. Chain P. S. (2014). Complete genome assembly of *Enterococcus faecalis* 29212, a laboratory reference strain. *Genome Announc.* 2 e968–e914. 10.1128/genomeA.00968-14 PMC4175211 25291775

[B42] Molander A. Reit C. Dahlen G. Kvist T. (1998). Microbiological status of root-filled teeth with apical periodontitis. *Int. Endod. J.* 31 1–7. 10.1046/j.1365-2591.1998.t01-1-00111.x 9823122

[B43] Oppenheimer-Shaanan Y. Steinberg N. Kolodkin-Gal I. (2013). Small molecules are natural triggers for the disassembly of biofilms. *Trends Microbiol.* 21 594–601. 10.1016/j.tim.2013.08.005 24080023

[B44] Paganelli F. L. Willems R. J. Leavis H. L. (2012). Optimizing future treatment of enterococcal infections: attacking the biofilm? *Trends Microbiol.* 20 40–49. 10.1016/j.tim.2011.11.001 22169461

[B45] Parsek M. R. Singh P. K. (2003). Bacterial biofilms: an emerging link to disease pathogenesis. *Annu. Rev. Microbiol.* 57 677–701. 10.1146/annurev.micro.57.030502.090720 14527295

[B46] Raad I. Chatzinikolaou I. Chaiban G. Hanna H. Hachem R. Dvorak T. (2003). In vitro and ex vivo activities of minocycline and EDTA against microorganisms embedded in biofilm on catheter surfaces. *Antimicrob. Agents Chemother.* 47 3580–3585. 10.1128/AAC.47.11.3580-3585.2003 14576121 PMC253809

[B47] Ramos I. Dietrich L. E. Price-Whelan A. Newman D. K. (2010). Phenazines affect biofilm formation by *Pseudomonas aeruginosa* in similar ways at various scales. *Res. Microbiol.* 161 187–191. 10.1016/j.resmic.2010.01.003 20123017 PMC2886020

[B48] Ricucci D. Siqueira J. F. Jr. (2010). Biofilms and apical periodontitis: study of prevalence and association with clinical and histopathologic findings. *J Endod* 36 1277–1288. 10.1016/j.joen.2010.04.007 20647081

[B49] Romero D. Kolter R. (2011). Will biofilm disassembly agents make it to market? *Trends Microbiol.* 19 304–306. 10.1016/j.tim.2011.03.003 21458996 PMC3750235

[B50] Sanchez C. J. Jr. Prieto E. M. Krueger C. A. Zienkiewicz K. J. Romano D. R. Ward C. L. (2013). Effects of local delivery of d-amino acids from biofilm-dispersive scaffolds on infection in contaminated rat segmental defects. *Biomaterials* 34 7533–7543. 10.1016/j.biomaterials.2013.06.026 23831189

[B51] She P. Chen L. Liu H. Zou Y. Luo Z. Koronfel A. (2015). The effects of d-Tyrosine combined with amikacin on the biofilms of *Pseudomonas aeruginosa*. *Microb. Pathog.* 86 38–44. 10.1016/j.micpath.2015.07.009 26188263

[B52] Shen Y. Stojicic S. Haapasalo M. (2010). Bacterial viability in starved and revitalized biofilms: comparison of viability staining and direct culture. *J. Endod.* 36 1820–1823. 10.1016/j.joen.2010.08.029 20951294

[B53] Shlezinger M. Houri-Haddad Y. Coppenhagen-Glazer S. Resch G. Que Y. A. Beyth S. (2016). Phage therapy: a new horizon in the antibacterial treatment of oral pathogens. *Curr. Top. Med. Chem.* 10.2174/1568026616666160930145649 [Epub ahead of print]. 27770768

[B54] Singh P. K. Parsek M. R. Greenberg E. P. Welsh M. J. (2002). A component of innate immunity prevents bacterial biofilm development. *Nature* 417 552–555. 10.1038/417552a 12037568

[B55] Stewart P. S. (2002). Mechanisms of antibiotic resistance in bacterial biofilms. *Int. J. Med. Microbiol.* 292 107–113. 10.1078/1438-4221-00196 12195733

[B56] Tay C. X. Quah S. Y. Lui J. N. Yu V. S. Tan K. S. (2015). Matrix metalloproteinase inhibitor as an antimicrobial agent to eradicate *Enterococcus faecalis* biofilm. *J. Endod.* 41 858–863. 10.1016/j.joen.2015.01.032 25814242

[B57] Tsume Y. Incecayir T. Song X. Hilfinger J. M. Amidon G. L. (2014). The development of orally administrable gemcitabine prodrugs with D-enantiomer amino acids: enhanced membrane permeability and enzymatic stability. *Eur. J. Pharm. Biopharm.* 86 514–523. 10.1016/j.ejpb.2013.12.009 24361461 PMC4243704

[B58] Vlamakis H. Chai Y. Beauregard P. Losick R. Kolter R. (2013). Sticking together: building a biofilm the *Bacillus subtilis* way. *Nat. Rev. Microbiol.* 11 157–168. 10.1038/nrmicro2960 23353768 PMC3936787

[B59] Wang Q. Q. Zhang C. F. Chu C. H. Zhu X. F. (2012). Prevalence of *Enterococcus faecalis* in saliva and filled root canals of teeth associated with apical periodontitis. *Int. J. Oral Sci.* 4 19–23. 10.1038/ijos.2012.17 22422085 PMC3412659

[B60] Yu C. Wu J. J. Contreras A. E. Li Q. L. (2012). Control of nanofiltration membrane biofouling by *Pseudomonas aeruginosa* using D-tyrosine. *J. Membr. Sci.* 423 487–494. 10.1016/j.memsci.2012.08.051

[B61] Zapata R. O. Bramante C. M. De Moraes I. G. Bernardineli N. Gasparoto T. H. Graeff M. S. (2008). Confocal laser scanning microscopy is appropriate to detect viability of *Enterococcus faecalis* in infected dentin. *J. Endod.* 34 1198–1201. 10.1016/j.joen.2008.07.001 18793919

[B62] Zhang C. Du J. Peng Z. (2015). Correlation between *Enterococcus faecalis* and persistent intraradicular infection compared with primary intraradicular infection: a systematic review. *J. Endod.* 41 1207–1213. 10.1016/j.joen.2015.04.008 26015157

