# Supplementary material for: Eradication of Enterococcus faecalis Biofilms on Human Dentin
Source: Front Microbiol. 2016 Dec 26;7:2055. doi: 10.3389/fmicb.2016.02055 (PMC5183576; doi:10.3389/fmicb.2016.02055)
Supplement: Supplementary file 1 [file Image_1.PDF]

Eradication of *Enterococcus faecalis* biofilms on human dentin

Supporting Figures

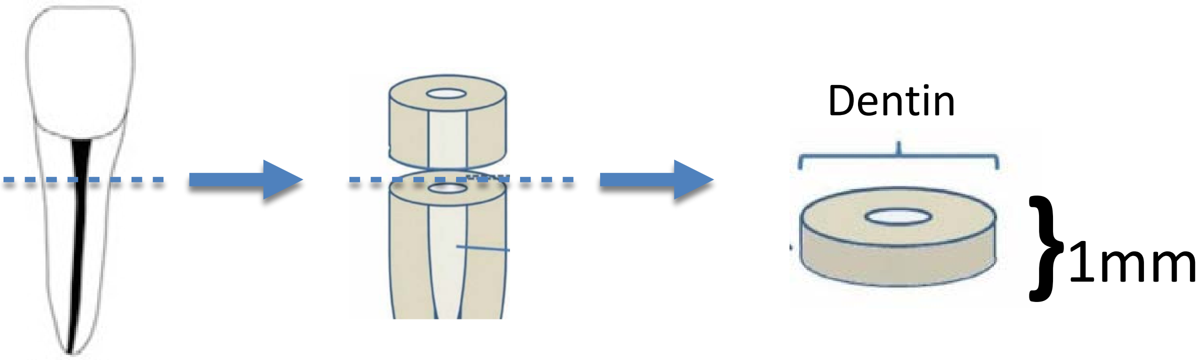

Figure S1. Preparation of dentin slabs. For details refer to materials and methods

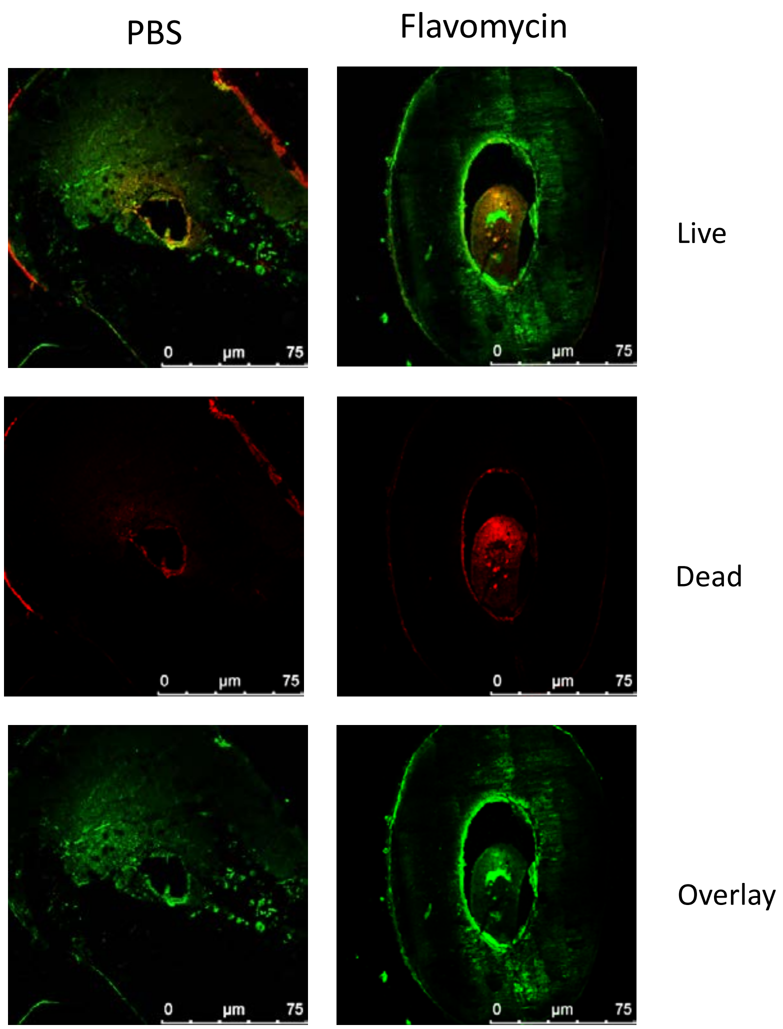

Figure S2. Flavomycin does not affect pre-established biofilms of *E. faecalis* on human dentin disks. Single colony of Strain 29212 was grown at 37 °C with shaking in liquid TSB-glucose media to a mid-logarithmic stage. Cells were diluted 1:100 into fresh media in polystyrene plates, containing a fixed dentin disk. Following 24 hours of incubation cells were applied with the following solutions, PBS (PBS), or flavomycin (2 µg/ml) for 2 hrs. Cells were washed, stained with BacLight Bacterial Viability kit and imaged as described in materials and methods.
